# FREQUENT CAUSES OF 30-DAY READMISSION FOLLOWING A FIRST EPISODE OF HEART FAILURE IN OLDER ADULTS

**DOI:** 10.1093/geroni/igac059.2535

**Published:** 2022-12-20

**Authors:** Ali Vaeli Zadeh, Andrew Crawford, Alan Wong, Elias Collado, Joshua Larned

**Affiliations:** Holy Cross Health-Jim Moran Cardiovascular Research Institute, Fort Lauderdale, Florida, United States; Holy Cross Health-University of Miami, Fort Lauderdale, Florida, United States; Holy Cross Health-University of Miami, Fort Lauderdale, Florida, United States; Holy Cross Health-Jim Moran Cardiovascular Research Institute, Fort Lauderdale, Florida, United States; Holy Cross Health- University of Miami, Miami, Florida, United States

## Abstract

Older adults with heart failure (HF) have a greater propensity to experience poor clinical outcomes, hospital readmission, and functional decline. It is important that healthcare workers are aware of the comorbidities that may lead to poor outcomes. A deeper understanding of potential causes of readmission will help in adequate prevention. Employing an all-payers claims database, we aimed to examine the diagnoses related to 30-day readmission in patients aged 65-75 with an Elixhauser-Comorbidity Index score < 4 upon the first episode of HF. Exclusion criteria included in-hospital death, readmissions for the same condition, and discharge against medical advice. Using ICD codes 9-10, a cohort of 323,678 patients (mean age: 70, SD: 3.22; male: 60%) with the first episode of HF between 2015-2019 were identified. We found 7,729 (2.4%) readmissions within 30 days and examined the frequency of 118 diagnoses as the primary cause. Cardiovascular etiologies including HF, hypertension, arrhythmias, and coronary artery disease were the most prevalent causes of readmission (40.64%), with HF being the most frequent (13.25%). Non-Cardiovascular causes included pulmonary (5.94%), sepsis (7%), renal (3.8%), and cerebrovascular (1.58%). Metabolic, liver, gastrointestinal diseases, and other infections accounted for 19.32%. Understanding the diagnoses related to readmissions in different populations is crucial for improving care, conducting research, and lowering costs. We focused on the first episode of HF in a relatively healthy older population to control for confounding factors. Further studies considering demographics, HF characteristics, and baseline comorbidities are needed.

